# Comparison of SPECT bone scintigraphy with MRI for diagnosis of meniscal tears

**DOI:** 10.1186/1471-2385-5-2

**Published:** 2005-04-14

**Authors:** Mohammad-naghi Tahmasebi, Mohsen Saghari, Masoud Moslehi, Ali Gholamrezanezhad

**Affiliations:** 1 MD, Orthopedic Department, Shariati hospital, Tehran University of medical sciences, Northern Kargar St. 14114, Tehran, Iran; 2 MD, Nuclear Medicine Research Center, Shariati hospital, Tehran University of medical sciences, Northern Kargar St. 14114, Tehran, Iran

## Abstract

**Background:**

Scintigraphy has been considered as competitive to MRI, but limited data are available on the accuracy of single photon emission tomography (SPECT) compared with MRI for the assessment of meniscal tears. Our objective was to assess the value of SPECT in comparison to MRI.

**Methods:**

Between January 2003 and March 2004, sixteen patients were studied with both modalities and the accuracy rates of SPECT scan results, and MRI findings in the diagnosis of meniscal tears were compared. Arthroscopy was the gold standard.

**Results:**

The respective sensitivity rate, specificity rate, and positive and negative predictive accuracies of MRI were 89%, 94%, 93%, and 79% and for SPECT those were 78%, 94%, 94%, and 88%. There was good agreement on the presence or absence of tears between two modalities (κ statistic = 0.699).

**Conclusion:**

SPECT and MRI are both valuable imaging techniques. SPECT is a useful alternative when MRI is unavailable or unsuitable and it is beneficial when more possible accuracy is desired (such as when MRI results are either inconclusive or conflict with other clinical data).

## Background

In diagnosing meniscal tears, MRI is a sensitive and specific tool and has become the procedure of choice in these affections. In fact, MRI is the most commonly used noninvasive imaging method for diagnosing meniscal tears, but its limits are also acknowledged [1].

On the other hand, although it is not widely carried out in clinical practice, nuclear medicine procedures have also been used in diagnosing meniscal tears and some authors have demonstrated the usefulness of SPECT in the assessment of knee injuries [2-8].

In particular, recent data [9] have demonstrated a higher specificity and accuracy for ^99m^Tc MDP SPECT than those for MRI. As a result, scintigraphy has been considered as competitive to MRI, and also it could give complementary informations which are commonly derived from scintigraphy but not available from MRI.

There are not so much published works concerning meniscal tears and bone SPECT [10-14]. Only few studies have compared bone scintigraphy and MR imaging and limited data are available on the accuracy of SPECT compared with that of MRI for the assessment of meniscal tears. Therefore, it was emphasized that further work should be undertaken to evaluate the role of SPECT as a screening test for the evaluation of knee disorders.

This study reports the results of a recent prospective evaluation of MRI and SPECT bone scintigraphy and compares them with the results of arthroscopy as the gold standard test for the diagnosis of meniscal tears.

## Methods

### Patients

Between January 2003 and March 2004 sixteen consecutive patients (13 men, 3 women), aged 15–52 yr (31 ± 10 yr, Mean ± S.D.), who were referred to our orthopedic surgeon were entered in this prospective study. Subjects were selected on the basis of positive history and clinical signs suggestive of meniscal tears. MRI and SPECT bone scintigraphy of both knees were obtained from all subjects. The time interval between the SPECT and MRI examinations was 1 to 3 weeks (mean time interval, 2.4 weeks). None of the patients had trauma or additional invasive therapeutic interventions between the SPECT and MRI scans. Patients also underwent arthroscopy of the affected knees.

### MRI

All studies were performed using a scanner (IGE Medical Systems, Signa Herza, Milwaukee, WI) with a 1.5 Tesla magnet. The knees were placed in an extended position with approximately 15° of external rotation. The imaging protocol included sagittal multiecho (repetition time msec/echo time msec, 2,500–3,600/20–120), coronal T1-weighted (600/12), coronal multiecho (2,500–3,000/17–119), and transverse gradient-echo or turbo T2-weighted sequences with a slice thickness of 4.5 mm, no interslice gap, and a matrix of 256 × 256. MRI results were reported by a radiologist experienced in MRI of the knees.

### Bone scintigraphy

A commercial MDP preparation (Myoview; Amersham International) was used. The labeling and quality control procedures were performed according to the manufacturer's instructions.

Scans were performed on a Vertex dual head ADAC camera. All patients received 750 MBq (20 mCi) ^99m^Tc methylene diphosphonate (^99m^Tc-MDP) by injection, and 3 hours later, anterior, posterior, medial and lateral views of both knees were obtained. SPECT was performed after securing the knees with a band around the tibiae and the legs straightened, with the same dual-head gamma camera, equipped with a pair of low energy, high resolution collimators. Images were acquired in a 128 × 128 matrix at 64 steps, 40 s each step. Data were processed by back projection and filtered by Hanning 0.8 filter. Images were reconstructed and displayed in all three orthogonal planes. Two experienced nuclear medicine physicians familiar with knee SPECT scans interpreted the findings of knee SPECT and the final diagnoses were reached by consensus. Blinded to other informations both MRI and bone SPECT were reported as definite or probable meniscal tear (positive) and normal or non-specific (negative). The positive criterion for meniscal tears was tibial plateau activity on the planar image with at least a half crescent of peripheral tibial plateau uptake on SPECT[12]. Other abnormal scintigraphic patterns were considered as non-specific. The positive criteria for meniscal tears in the MRI were abnormal morphology of the meniscus on one or more MR images and/or abnormal increased signal in that area on fat saturated proton density or T2-weighted images. Arthroscopy was performed by our experienced arthroscopist who already knows the results of the MRI and SPECT bone scans at the time of the examination.

### Statistical evaluation

Results were analyzed on a per-meniscus basis. Using arthroscopy as a gold standard, the results of each modality were analyzed for sensitivity, specificity, negative predictive value and positive predictive value. Differences between these performance indices in the two modalities were evaluated with the McNemar test. *P *< 0.05 was considered to be statistically significant.

All patients gave informed consent to participate in this study, which was approved by the committee on ethics at the faculty of medicine, university of Tehran.

## Results

A total of 32 menisci, including 16 left and 16 right menisci, in 16 patients (table 1) were assessed. According to the arthroscopic results, tears were present in 18 (56%) menisci (table 2), of these thirteen tears were in the medial menisci, five tears were in the lateral menisci, ten tears were in the left menisci and eight tears were in the right menisci.

**Table 1:** Patients' characteristics and results of MRI, SPECT and Arthroscopy.

Patient no.	Sex	Age	MRI	SPECT	Arthroscopy
1	M	34	LM	LM	LM
2	M	15	NL	NL	NL
3	M	19	RM	RM	RM
4	F	52	LM	LM/LL	LM/LL
5	M	45	RM	RM	RM
6	M	35	LM	LM	LM
7	M	30	LL	LL	LL
8	F	50	RM	D.I.U	RM
9	M	26	RM/RL	RL	RM/RL
10	M	24	RM	RM	RM
11	M	26	LM	LM/LL	LM
12	M	28	RM	RM	RM
13	M	25	LL	NL	LM
14	M	30	LM/LL	LM	LM/LL
15	F	32	RL	RL	RL

16	M	21	LM	LM	LM

* M = Male, F = Female, D. I. U = Diffusely Increased Uptake, RL = Right Lateral, RM = Right Medial, LL = Left Lateral, LM = Left Medial, NL = Normal.

**Table 2:** Arthroscopy results by meniscus.

Meniscus	Positive	Negative	Total
Left/Medial	7	1	8
Left/Lateral	3	5	8
Right/Medial	6	2	8
Right/Lateral	2	6	8

Total	18	14	32

By consensus, observers detected 15 meniscal tears at SPECT readings, of which one was falsely positive (table 3). One knee showed generalized increased uptake on bone SPECT images, in which the exact anatomical location of the pathologic process could not be determined and this finding was categorized as false-negative for meniscal tear. Overall there were only four false-negative SPECT scans.

**Table 3:** Bone SPECT readings by meniscus.

Meniscus	Positive	Negative	Total
Left	9	7	16
Right	6	10	16

Total	15	17	32

MRI detected 17 tears (table 4). However one of them was false positive. Two meniscal tears were missed by MRI. Among the tears detected on SPECT images, only one tear was not detected by MRI. On the other hand, three meniscal tears were not depicted on SPECT images, which were detectable on MR images.

**Table 4:** MRI reading by meniscus.

Meniscus	Positive	Negative	Total
Left	9	7	16
Right	8	8	16

Total	17	15	32

Overall, MRI had a positive predictive value of 94% (16/17) and SPECT had 93% (14/15). MRI had a negative predictive value of 88% (15/17) and SPECT had 79% (15/19).

Table 5 shows the sensitivities, specificities, positive predictive values, and negative predictive values for each imaging modality. The two-tailed ***p ***value of these differences equals 0.683, which by conventional criteria, is considered to be not statistically significant. MRI and SPECT results were further compared on another per meniscus basis according to whether SPECT findings were positive or negative (table 6). In a total of 17 knees which underwent imaging with both modalities, meniscal tear was found in 17 menisci with MRI and in 15 menisci with SPECT. Concordant positive results were reported in 13 menisci. In four menisci, MRI depicted additional tears. The κ value, as a measure for agreement between SPECT and MRI, revealed that despite differences between methods in sensitivity and specificity for the detection of meniscal tears, there was still good overall agreement (κ statistic = 0.699, with standard error = 0.101).

**Table 5:** A comparison of diagnostic ability of SPECT and MRI in diagnosis of meniscal tears.

	MRI	SPECT
Sensitivity	89%	**78%**
Specificity	94%	**94%**
Positive predictive value	93%	**94%**

Negative predictive value	**79%**	**88%**

**Table 6:** Summary of knee SPECT and MRI results.

Test Results	No. of menisci
Positive SPECT vs positive MRI	13
Positive SPECT vs Negative MRI	2
Negative SPECT vs Positive MRI	4

Negative SPECT vs Negative MRI	13

## Conclusion

MRI has become the radiologic procedure of choice for the diagnosis of meniscal tears. Recently SPECT also has been used for assessment of knee pathologies and has been documented to have a higher sensitivity than MRI. One of the most widely referenced studies is that of Ryan PJ et al. [9], in which 100 patients with undiagnosed knee pain were studied by clinical examination, MRI, SPECT bone scintigraphy and arthroscopy. The authors found the accuracy of MRI and SPECT in detecting meniscal tears to be comparable. Using arthroscopy as a gold standard, both MRI and SPECT showed high diagnostic ability to detect meniscal tears, with respective sensitivity rate, specificity rate, and positive and negative predictive accuracies of 80%, 71%, 84% and 71% for MRI and 84%, 80%, 88% and 76% for SPECT. Some meniscal tears were detected by MRI alone (n = 5), or SPECT alone (n = 8). These authors concluded that SPECT bone scintigraphy is a suitable alternative to MRI to detect meniscal tears. It was also noted that the comparable diagnostic ability of SPECT bone scintigraphy implies that it can be used successfully when MRI is unavailable or unsuitable.

Even-Sapir and colleagues reported on 94 patients with suspected ACL/meniscal tear, or both who underwent SPECT followed by arthroscopy (n = 74), magnetic resonance imaging (n = 37), or both [15]. Tears of the medial meniscus were diagnosed by arthroscopy in 43 patients. SPECT images detected increased uptake in the medial tibial plateau with a positive predictive value of 78% and a negative predictive value of 83%. These authors suggest that bone SPECT is valuable in acute knee trauma for assessment of ACL, meniscal tears or both and for detection of associated bone injury.

In another study of patients with chronic knee pain Collier et al., found a high sensitivity of SPECT for the detection of meniscal tears, although specificity was less good [16].

In one of the initial studies, Murray et al. [11] found a SPECT sensitivity of 88% and specificity of 87% in patients with acute knee pain. They concluded that with respect to meniscal tears a negative bone scan can obviate the need for arthroscopy.

In the recent study done by Vellala RP et al. [17] the role of SPECT bone scan for the diagnosis of knee lesions in routine clinical practice was evaluated. Fourty consecutive case records were examined in patients who underwent a SPECT scan prior to knee arthroscopy in routine clinical practice. The accuracy of clinical examination, SPECT scan results, and arthroscopic findings (as the gold standard) in diagnosing knee lesions were compared. The sensitivity of SPECT scans in detecting medial meniscal and lateral meniscal lesions was 77% and 14%, respectively. The specificities for the same structural lesions were high at 89% and 94%, respectively. The authors concluded that SPECT bone scan appears to be useful in the diagnosis of knee pathology in routine practice and in selecting patients for arthroscopy, especially most useful for the diagnosis of medial meniscal tears.

Our results are not very different from the above-mentioned researches. The present prospective study demonstrated that MRI was only slightly superior to SPECT for detection of meniscal tears. However, the difference did not reach statistical significance. In our study SPECT revealed the majority of lesions seen on arthroscopy and MRI. Also there was a tear that was missed by MRI but adequately diagnostic by SPECT. Similarly there were some tears in which SPECT was negative but MRI showed the tears. It is not yet well determined that why some tears are missed by one modality and are detected by the other. However, it seems that in the presence of high clinical suspicion and negative MRI results (as the primary modality), SPECT can be helpful with detecting MRI negative tears. Despite of this fact, the detection of more tears at SPECT compared with MRI, did not lead to altered decision for treatment (i.e. conservative vs. arthroscopic treatment). The major reason is that the difference in detection of lesion was predominantly in patients in whom another MRI-positive tear was detected. These patients are usually candidate for arthroscopy and therefore differences in the number of tears do not influence management.

In general, the findings of our study and that of previous studies suggest that examination with SPECT as well as with MRI can be used as a basis for the assessment of patients suspected for meniscal tears. However, both MRI & SPECT have various relative advantages and disadvantages. In general, SPECT is less costly than MRI because it involves lower capital equipment costs. SPECT is also widely available. The major limitation with the use of SPECT is the radiation exposure, the potential harm of which is poorly understood.

MRI also has some advantages over SPECT of the knees too. Most significantly, no ionizing radiation is used. MRI also has some important drawbacks, however. In most regions it is a more expensive than SPECT and has more contraindications and scheduling difficulties. Some authors concluded that MRI, except in certain circumstances, is an expensive and unnecessary diagnostic test in patients with suspected meniscal and ACL pathology (may be due to many false positive MRI reports) [18,19].

These facts in addition to all of the above-mentioned research results indicate that SPECT and MRI are both valuable advanced imaging techniques but the absence of radiation exposure may make MRI preferable for the workup of patients suspected of having meniscal tears. Therefore it seems that all patients suspected for meniscal tears can be evaluated with MRI. However, SPECT has clear advantages when more possible accuracy is desired when MRI results are either inconclusive or conflict with other clinical data (i.e. SPECT should be performed if MRI is negative but there are clinical evidences of meniscal tear). SPECT may be available alternative when MRI is unavailable or unsuitable. This approach must be addressed in larger series of patients and a larger prospective study is currently being performed to confirm these data and approach.

## Competing interests

The author(s) declare that they have no competing interests.

## Authors' contributions

MNT participated in the design of the study and carried out the arthroscopies. MS participated in the interpretation of the scintigraphic results. MM participated in its design and coordination, supervised the acquisition process and participated in the interpretation of the scintigraphic results. AG supervised the acquisition process, interpreted the scintigraphic results, performed the statistical analysis and drafted the manuscript. All authors read and approved the final manuscript.

**Figure 1:**
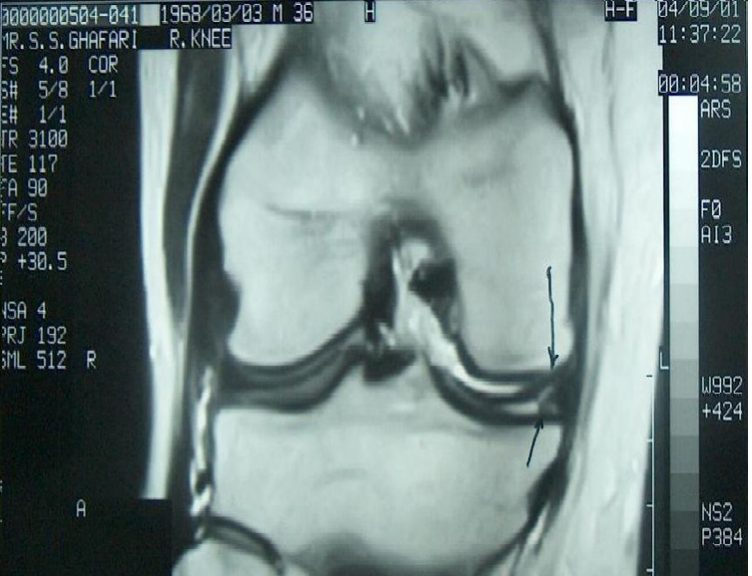
Magnified coronal T2-weighted MRI image of the right knee showing a medial meniscal tear.

**Figure 2:**
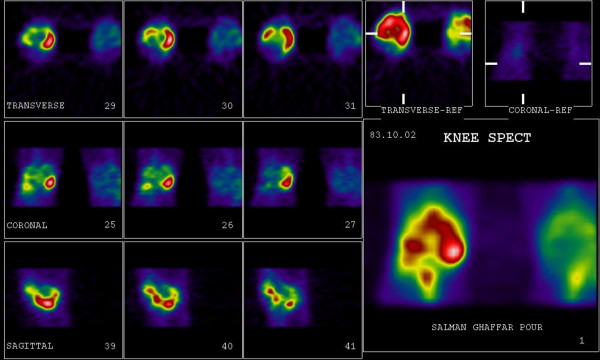
SPECT images of the same patient (presented in Figure 1.) showing a crescent of increased activity in the medial tibial plateau, which is scintigraphically characteristic feature of a meniscal tear.

## Pre-publication history

The pre-publication history for this paper can be accessed here:


